# Supplementary material for: Application of Deep Learning on Single-cell RNA Sequencing Data Analysis: A Review
Source: Genomics Proteomics Bioinformatics. 2022 Dec 14;20(5):814–35. doi: 10.1016/j.gpb.2022.11.011 (PMC10025684; doi:10.1016/j.gpb.2022.11.011)
Supplement: Supplementary Figure 1 — Pipeline of study inclusion and exclusion for survey We first isolated documents using a PubMed search using the phrases (“single cell” AND (“deep learning OR autoencoder”)) AND “sequencing”. Specifically, we conducted the search on April 1, 2021 and Jan 5, 2022, respectively, and combined the results together, because we expected to incorporate studies that were recently published. We filtered papers prior to 2019 to include the most recent work related to the use of deep learning in the field. This yielded 192 papers. Second, we also conducted search in other resources like Google Scholar or identified articles based on citing relationships among papers using a snow-ball strategy, which yielded 43 papers. After deduplicating, we obtained a total of 226 articles. All articles were evaluated manually, and studies were excluded if: (1) the main focus is not to improve scRNA-seq data analysis; (2) the model involved is not a deep learning algorithm; and (3) the paper is not a research article. Finally, a total of 98 studies were included for survey. [file mmc1.pdf]

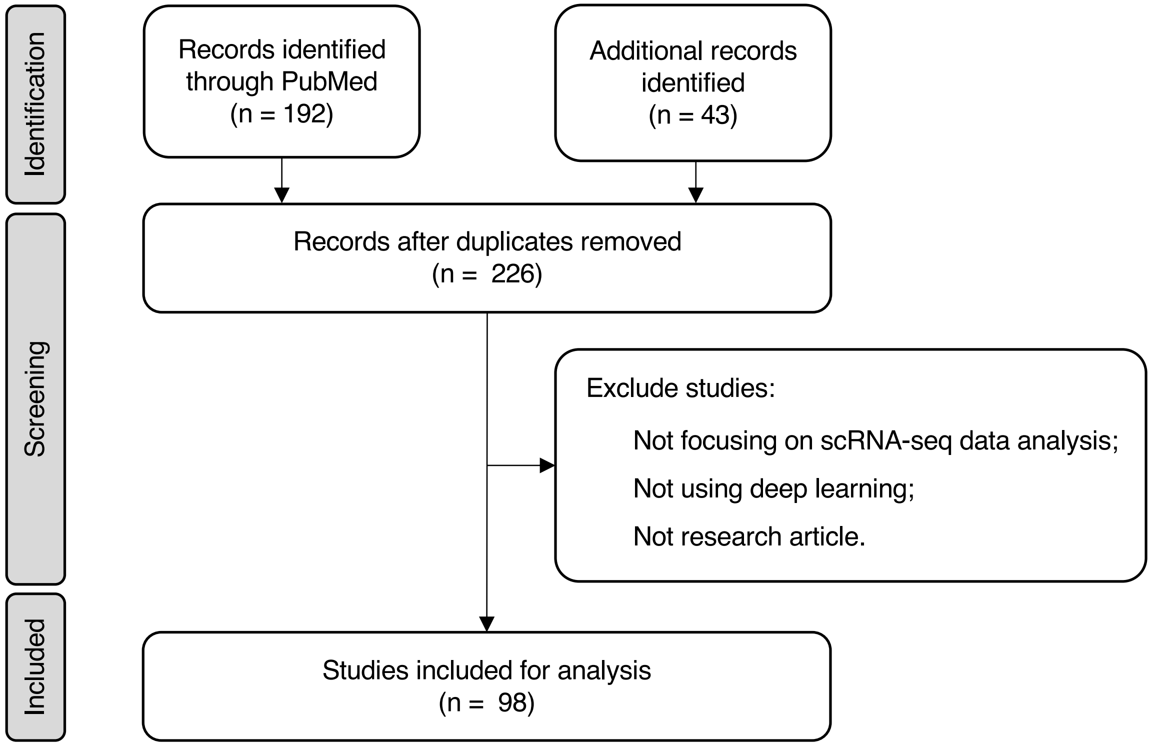
Identification
Screening
Included
Records identified through PubMed (n = 192)
Additional records identified (n = 43)
Records after duplicates removed (n = 226)
Exclude studies:
Not focusing on scRNA-seq data analysis;
Not using deep learning;
Not research article.
Studies included for analysis (n = 98)
